# Acupotomy for the treatment of lumbar spinal stenosis: A protocol for a systematic review and meta-analysis

**DOI:** 10.1097/MD.0000000000014160

**Published:** 2019-01-18

**Authors:** Chan-Young Kwon, Sang-hoon Yoon, Boram Lee, Jungtae Leem

**Affiliations:** a Chung-Yeon Central Institute, Gwangju, Republic of Korea; b Department of Clinical Korean Medicine, Graduate School, Kyung Hee University, Seoul, Republic of Korea; c Chung-Yeon Korean Medicine Hospital, Gwangju, Republic of Korea; d Department of Korean Medicine, Kyung Hee University Korean Medicine Hospital at Gangdong, Seoul, Republic of Korea; e Dongshin Korean Medicine Hospital, Seoul, Republic of Korea.

**Keywords:** acupotomy, protocol, spinal stenosis, systematic review

## Abstract

**Introduction:**

This systematic review protocol describes the methods that will be used to evaluate the efficacy and safety of acupotomy for the treatment of lumbar spinal stenosis.

**Methods and analysis:**

The following electronic databases will be searched until December 2018 without language or publication status restrictions: five English databases, that is Medline, the Cochrane Central Register of Controlled Trials (CENTRAL), EMBASE, Allied and Complementary Medicine Database (AMED), and Cumulative Index to Nursing and Allied Health Literature (CINAHL); three Korean databases, that is Oriental Medicine Advanced Searching Integrated System (OASIS), Research Information Service System (RISS), and Korea Citation Index (KCI); and three Chinese databases, that is China National Knowledge Infrastructure (CNKI), Wanfang Data, and VIP. Any clinical, randomized controlled trials using acupotomy for lumbar spinal stenosis treatment will be included. Changes in the degrees of pain and function will be assessed as primary outcomes. The total effective rate, changes in quality of life, adverse events, and amount of rescue medication used will be evaluated as secondary outcomes. Two independent researchers will perform study selection, data extraction, and risk of bias assessment. If applicable, a meta-analysis will be performed using RevMan version 5.3, with the results expressed as risk ratios or mean differences with 95% confidence intervals. According to a heterogeneity test or the number of studies included, fixed effects or random effects model will be used. The risk of bias tool from the Cochrane group will be used to evaluate the methodological quality of the included studies.

**Ethics and dissemination:**

Ethical approval is not required because individual patient data will not be included in this study. The findings of this systematic review will be disseminated through a peer-reviewed publication or conference presentations.

**PROSPERO registration number:**

CRD42018116567.

## 1 Introduction

Spinal stenosis refers to an abnormal narrowing of spinal canal or neural foramen, which may cause clinical symptoms secondary to compression of the spinal cord or nerve roots. Lumbar spinal stenosis (LSS) is a common degenerative condition that becomes more prevalent with age and often leads to spinal surgery.^[1,2]^ A longitudinal population-based cohort study revealed that the prevalence of LSS, with an interval of 10 mm between the intervertebral disc and ligamentum flavum, was 4% in people less than 40 years old, and increased with age, reached approximately 14% in the age group of 60 and over.^[3]^ Although not all of the radiographically diagnosed LSS patients present with clinical signs,^[4]^ symptomatic LSS can present as low back pain (LBP) with or without leg symptoms, disability, neurogenic claudication, and locomotive syndrome.^[5,6]^ During a cohort study of the natural clinical progression of LSS, patients’ symptoms were classified as improved, unchanged, or exacerbated over the mean follow-up period of 11 years. Each group accounted for 30% of LSS patients, and half of the exacerbated group received surgical treatment.^[7]^

Surgical intervention, physical therapy, and exercise are standard treatments for LSS. The surgical rate for LSS is increasing rapidly^[1,8]^; however, recent surgical interventions such as spinal decompression surgery, simple spinal fusion, and complex spinal fusion have been associated with high postoperative complications^[9,10]^ and relatively low long-term satisfaction.^[11,12]^ More recently, the issue of so-called “failed back surgery syndrome” has emerged, presenting challenges and hesitation in the treatment of LBP patients.^[13]^ Therefore, minimally invasive operative management of LSS has gained popularity.^[14,15]^

Acupotomy, also referred to as mini-scalpel needle or needle-knife,^[16]^ is one complementary and integrative medicine (CIM) modality that modernizes acupuncture by combining conventional acupuncture needle and small-knife. It has been used as a tool for minimally invasive operative management for decades. The origin of the treatment is “Nine Classical Needles” from the era of Huangdi's Internal Classic (Huangdi's Internal Classic, *Huang Di Nei Jing*); the treatment was developed into a modernized tool, acupotomy, by Zhu Hanzhang in 1976.^[17]^ Nowadays, acupotomy is widely used for musculoskeletal conditions, especially in China and Republic of Korea. Clinical evidence suggests that this treatment can relax muscular spasm and relieve compressed nerves and vessels by using the small-knife to detach taut muscle bands.^[18]^ Moreover, in a recent network meta-analysis study of the effects of various types of acupuncture in myofascial pain syndrome, acupotomy was a superior modality for improving the pressure pain threshold when compared to manual acupuncture, electro-acupuncture, dry-needling, acupuncture point injection, and fire-needle.^[19]^ Similarly, it has been reported that acupotomy may improve pain and quality of life of LSS patients.^[20]^

Because it combines the effect of minimally invasive operation^[14,15]^ using the small-knife with conventional acupuncture,^[21,22]^ acupotomy is a promising candidate for the treatment of LSS. However, there have been no systematic reviews assessing acupotomy for the treatment of LSS. In this review, we will investigate and critically review the current evidence on the efficacy and safety of acupotomy in LSS.

## 2 Methods and analysis

The protocol for this review has been registered in the International Prospective Register of Systematic Reviews (PROSPERO) (registration number, CRD42018116567) on December 5, 2018. If protocol amendments occur, the dates, changes, and rationales will be tracked in PROSPERO. This protocol was reported in accordance with the Preferred Reporting Items for Systematic Review and Meta-Analysis Protocols (PRISMA-P) 2015 statement^[23]^ and the Cochrane Handbook for Systematic Reviews of Interventions.^[24]^

### 2.1 Data sources and search strategy

The following databases will be searched comprehensively from their inception to December 2018: five English databases, that is, Medline, the Cochrane Central Register of Controlled Trials (CENTRAL), EMBASE, Allied and Complementary Medicine Database (AMED), and Cumulative Index to Nursing and Allied Health Literature (CINAHL); three Korean databases, that is Oriental Medicine Advanced Searching Integrated System (OASIS), Research Information Service System (RISS), and Korea Citation Index (KCI) and three Chinese databases, that is, China National Knowledge Infrastructure (CNKI), Wanfang Data, and VIP. The reference lists of the relevant articles will be searched, and a manual search on Google Scholar will be performed to identify additional trials. In addition, “gray literature” such as conference proceedings and theses will be allowed. No language restriction will be imposed.

The search terms will be composed of the disease term part (e.g., “lumbar spinal stenosis”) and the intervention term part (e.g., acupotomy or acupotomology or “needle knife” or needle-knife or needle-scalpel or miniscalpel or mini-scalpel or “stiletto needle” or “sword-like needle”). The search strategies in Medline database are shown in Table 1.

**Table 1:**
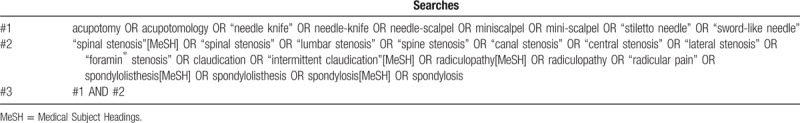
Search terms used in Medline.

### 2.2 Inclusion criteria

#### 2.2.1 Types of studies

We will include only randomized controlled trials (RCTs). Among them, we will exclude RCTs using a quasi-random method such as alternate allocation or allocation by birthdate. If the expression “randomization” is mentioned without the randomization methods, it will be included in this review. We will include both parallel and crossover studies. In crossover studies, we will use only first-phase data to calculate the effect size and to conduct meta-analysis. Other designs such as *in vivo*, *in vitro*, case reports, and retrospective studies will be excluded.

#### 2.2.2 Types of participants

We will include studies on patients diagnosed with LSS, regardless of sex, age, race, or severity and duration of disease. Studies will be excluded if the participants have other serious illnesses such as cancer, liver disease, or kidney disease.

#### 2.2.3 Types of interventions

We will only include studies using acupotomy as the sole experimental intervention. For control interventions, we will include wait-list, sham treatment, or active controls such as acupuncture, physiotherapy, and nerve block. Studies involving acupotomy combined with other therapies will be included if the other therapies are equally used in both experimental and control groups. We will exclude studies comparing different forms of acupotomy.

#### 2.2.4 Types of outcome measures

The primary outcome measures are (1) LBP symptoms measured by the visual analogue scale (VAS)^[25]^ and Japanese Orthopaedic Association (JOA) score^[26]^ and (2) functional outcomes measured by the Oswestry Disability Index (ODI).^[27]^

The secondary outcome measures are as follows:

(1) Total effective rate
  The total effective rate is a non-validated outcome measure that is processed secondarily according to certain evaluation criteria such as clinical symptom improvement or the improvement rates of other quantified outcomes. In the assessment of the total effective rate, participants are generally classified as “cured”, “markedly improved”, “improved”, or “non-responder” after treatment. The total effective rate is calculated consistently using the following formula: 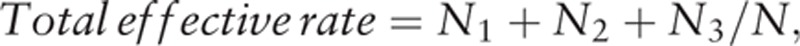

where *N*_1_, *N*_2_, *N*_3_, and *N* are the number of patients who are cured, markedly improved, improved, and who comprise the sample size, respectively.
(2) Health-related quality of life measured by such as 36-item short-form health survey (SF-36)^[28]^
(3) The incidence of adverse events
(4) Amount of rescue medication required.

### 2.3 Study selection

**Figure 1:**
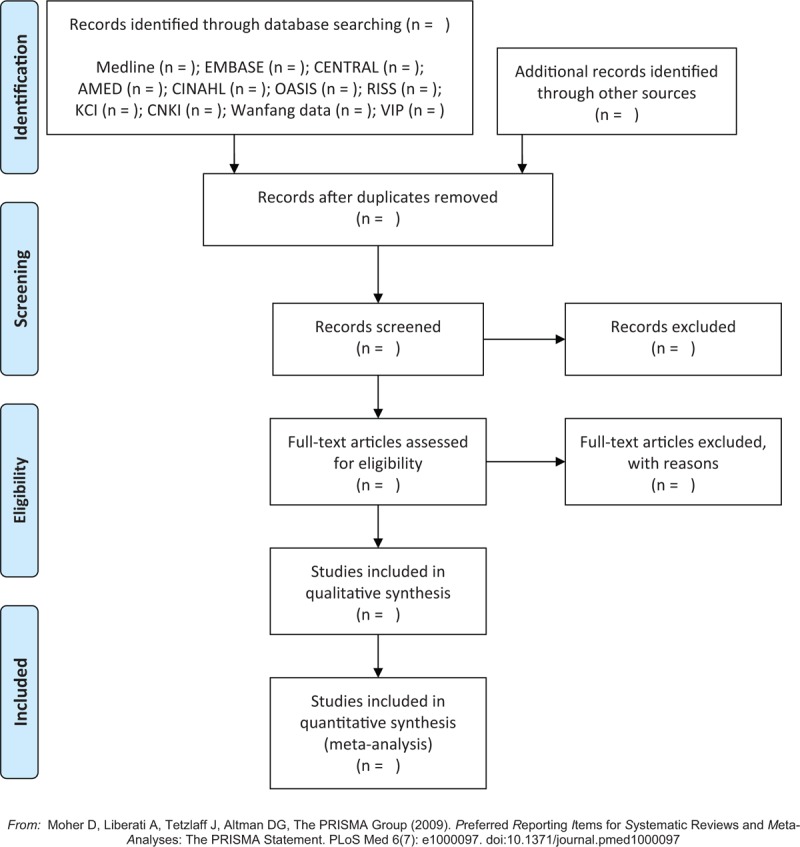
A PRISMA flow diagram of the literature screening and selection processes. AMED = Allied and Complementary Medicine Database, CENTRAL = Cochrane Central Register of Controlled Trials, CINAHL = Cumulative Index to Nursing and Allied Health Literature, CNKI = China National Knowledge Infrastructure, KCI = Korea Citation Index, OASIS = Oriental Medicine Advanced Searching Integrated System, RISS = Research Information Service System.

Two researchers (C-Y Kwon and B Lee) will conduct the study selection process according to the above criteria. After removing duplicates, we will evaluate the titles and abstracts of the searched studies for eligibility and then evaluate the full texts of the remaining studies for final inclusion. Any disagreement on study selection will be resolved through discussion with other researchers. We will report the study selection process according to the Preferred Reporting Items for Systematic Reviews and Meta-Analyses (PRISMA) guidelines (Fig. 1).^[29]^

### 2.4 Data extraction

Two researchers (C-Y Kwon and B Lee) will independently perform the data extraction using a standardized data collection form (Excel 2007, Microsoft, Redmond, WA, USA). Discrepancies will be resolved through discussion with other researchers.

The extracted items will include the first author's name; year of publication; country; approval of institutional review board; informed consent; sample size and number of dropouts; details about the participants, intervention, and comparisons; duration of the intervention; outcome measures; results; and adverse events. In accordance with the Standards for Reporting Interventions in Clinical Trials of Acupuncture (STRICTA), we will extract details of acupotomy such as stimulation site, needle type, anesthesia, and texture or sensation indicating proper procedure.^[30]^ We will contact the corresponding authors of the included studies via E-mail to request additional information if the data are insufficient or ambiguous.

### 2.5 Risk of bias assessment

Two researchers (C-Y Kwon and B. Lee) will independently assess the risk of bias of the included studies using the Cochrane group's risk of bias tool.^[31]^ The following domains will be assessed: random sequence generation, allocation concealment, blinding of participants and personnel, blinding of outcome assessments, completeness of outcome data, selective reporting, and other biases. If only the expression “randomization” (randomization) was mentioned without specifying the randomization methods, we will judge the study to be at high risk of bias in the random sequence generation domain. In addition, we will assess other bias domains with particular emphasis on baseline imbalance between experimental and control group characteristics such as mean age, sex, or disease severity, as these factors can cause bias in the estimation of the intervention effect. Each domain will be categorized into one of three groups: “low risk,” “unclear risk,” or “high risk.” We will present the evaluated results using Review Manager version 5.3 software (Cochrane, London, UK) in a full review. Disagreements between two researchers over the risk of bias will be resolved by discussion, with the participation of additional researchers.

### 2.6 Data analysis

We will perform the data analysis and synthesis using Review Manager version 5.3 software (Cochrane, London, UK). Descriptive analyses of the details of the participants, interventions, and outcomes will be conducted for all included studies. Meta-analysis will be performed across studies using the same types of intervention, comparison, and outcome measure. We will pool the continuous data using the mean difference or standardized mean difference with 95% confidence intervals (CIs) and the dichotomous data using the risk ratio with 95% CIs. Heterogeneity, in terms of effect measures between the studies, will be assessed using both the chi-squared test and the *I*-squared statistic. We will consider *I*-squared values ≥50% and ≥75% indicative of substantial and considerable heterogeneity, respectively. In the meta-analyses, we will use a random effects model when the heterogeneity is significant (an *I*-squared value ≥50%), while a fixed effects model will be used when the heterogeneity is non-significant. A fixed effects model will also be used when the number of studies included in the meta-analysis is less than five, where inter-study variance estimates have poor accuracy.^[32,33]^

### 2.7 Subgroup analysis

If the necessary data are available, we will conduct a subgroup analysis according to the following criteria: (1) the treatment period, (2) severity of LSS, and (3) type of active controls such as acupuncture, physiotherapy, and nerve block.

### 2.8 Sensitivity analysis

We will perform sensitivity analyses by excluding (1) studies with high risks of bias and (2) outliers that are numerically distant from the rest of the data, to identify the robustness of the meta-analysis result.

### 2.9 Assessment of reporting bias

If more than 10 trials are included in the meta-analysis, reporting biases, such as publication bias, will be assessed using funnel plots. When reporting bias is implied by asymmetry of funnel plot, we will attempt to explain possible reasons.

## 3 Ethics and dissemination

Ethical approval will not be needed because the data used in this systematic review will not be individual patient data, and there will be no concerns regarding privacy. The results will be disseminated by the publication of a manuscript in a peer-reviewed journal or presentation at a relevant conference.

## 4 Discussion

LSS is a leading cause of various medical and socioeconomic effects, especially in elderly patients.^[1–3]^ To date, minimally invasive approaches are one way to overcome the limitation of surgical treatment for LSS.^[14,15]^ Acupotomy, as a kind of minimally invasive technique in CIM, has been used to treat various musculoskeletal diseases including LSS.^[18–22]^ This treatment has the advantage of simultaneously achieving the effect of conventional acupuncture and releasing pressure on the intervertebral disc by micro-incision of lumbar extensor muscle.^[34]^

Animal studies reported that after acupotomy on the spine, the levels of nitric oxide synthase and beta-endorphin,^[35]^ or substance P, 5-hydroxytryptamine, interleukin-1β, interleukin-10, and transforming growth factor-α in peripheral blood^[36]^ were decreased, suggesting its anti-inflammatory and anti-nociceptive effects. Additionally, acupotomy was significantly superior to the electro-acupuncture in reducing the apoptotic cell number in the spondylosis rabbit model.^[37]^ In the same model study performed three years later, the ratio of Bcl-2/Bax mRNA was significantly higher than that of electro-acupuncture.^[38]^ It suggests that acupotomy can prevent continuous cell damage.

Likewise, a LBP guideline from the China Association of Acupuncture-Moxibustion recommended acupotomy for treating pathological changes in lumbar soft tissue.^[39]^ Moreover, some clinical evidence suggested that acupotomy may improve pain and quality of life of LSS patients.^[20]^ In summary, acupotomy may be an effective minimally invasive treatment for LSS, but no attempt has been made to review previous studies. Therefore, this systematic review will provide a comprehensive and critical evaluation of the efficacy and safety of acupotomy for LSS and elicit clinical implications.

We believe that our findings will encourage clinicians to support the evidence-based treatment of LSS with alternative techniques. In addition, we will analyze, in detail, the procedures of acupotomy used in the included studies. These data will help develop clinical research designs for acupotomy and be used in the application of acupotomy for the treatment of LSS. The development of new minimally invasive therapies such as acupotomy will address the problems associated with emerging surgical treatments and help reduce individual, societal, and national burden of LSS. Finally, our results will benefit LSS patients by offering a greater variety of treatment options.

## Acknowledgments

This study is supported by Chung-Yeon Medical Institute (Grant No. Research Program 2018). The funding source will have no input on the interpretation or publication of the study results.

## Author contributions

The study was conceptualized by CYK. The protocol was drafted by CYK, SHY, and BL. The search strategy was developed by CYK and BL. JL revised the manuscript, and all authors have read and approved the final manuscript. JL submitted the manuscript for publication.

**Conceptualization:** Chan-Young Kwon, Boram Lee.

**Funding acquisition:** Jungtae Leem.

**Methodology:** Chan-Young Kwon, Sang-hoon Yoon, Boram Lee.

**Writing – original draft:** Chan-Young Kwon, Boram Lee.

**Writing – review & editing:** Chan-Young Kwon, Sang-hoon Yoon, Boram Lee, Jungtae Leem.

Jungtae Leem orcid: 0000-0003-3300-5556.
